# The relationship between diabetes mellitus and attention deficit hyperactivity disorder: A systematic review and meta-analysis

**DOI:** 10.3389/fped.2022.936813

**Published:** 2022-09-29

**Authors:** Yuan Ai, Jing Zhao, Hanmin Liu, Jiao Li, Tingting Zhu

**Affiliations:** ^1^Department of Pediatrics, West China Second University Hospital, Sichuan University, Chengdu, China; ^2^Key Laboratory of Obstetric & Gynecologic and Pediatric Diseases and Birth Defects of Ministry of Education, Sichuan University, Chengdu, Sichuan, China

**Keywords:** ADHD, diabetes mellitus, risk factor, meta-analysis, child

## Abstract

**Background:**

This study aims to investigate the prevalence estimate of diabetes mellitus (DM) among people with attention deficit hyperactivity disorder (ADHD) as well as the prevalence of ADHD among those with DM. In addition, the impact of ADHD on glycemic control in patients with DM was also assessed using a systematic review and meta-analysis of currently available published data.

**Materials and methods:**

The PubMed, Embase, Web of Science, and PsycInfo databases were searched for potential studies. Two reviewers independently selected studies according to the inclusion and exclusion criteria. All pooled analyses were conducted using the random-effects models on Review Manager 5.3.

**Results:**

Seventeen observational studies were included. The pooled results showed an increase in the prevalence of DM among patients with ADHD versus those without ADHD [type 1 DM OR: 1.37 (95% CI: 1.17–1.61); type 2 DM OR: 2.05 (95% CI: 1.37–3.07)]. There was an overall 35% increase in the prevalence of ADHD among patients with type 1 DM [OR: 1.35 (95% CI: 1.08–1.73)]. Children with type 1 DM and ADHD had higher levels of hemoglobin A1c [standardized mean of differences: 0.67 (95% CI: 0.48–0.86)], and prevalence of hypoglycemic and ketoacidosis index compared with those without ADHD.

**Conclusion:**

Our study revealed the bidirectional associations between ADHD and DM. Patients with ADHD and type 1 DM comorbidities were more likely to have poorer diabetes control. More studies are needed to confirm this association and elucidate the underlying mechanism.

## Introduction

Attention deficit hyperactivity disorder (ADHD) is the most common neurodevelopmental disorder characterized by inattention and/or hyperactivity/impulsivity, leading to poor academic and social outcomes, with prevalence rates ranging from 3 to 7% (1, 2). In approximately 75% of childhood cases, ADHD symptoms can persist into adulthood in approximately 75% of childhood cases (3). The etiology of ADHD is not fully understood, and is considered to be the result of many factors. Patients with ADHD have a higher risk of migraines, autism spectrum disorder, and intellectual disability (4–6). These findings highlight the shared etiology of common neurological diseases. In addition, there is emerging evidence that patients with ADHD have a higher risk of developing metabolic syndrome. A recent meta-analysis of 95 studies reported that the pooled estimates of the prevalence of obesity and being overweight were 14.7% and 20.9%, respectively, in individuals with ADHD (7). A longitudinal cohort of 12,288 respondents found that childhood hyperactive/impulsive symptoms increased the risk of hypertension and obesity in adulthood (8).

Diabetes mellitus (DM) is a common metabolic disease characterized by elevated blood glucose levels. The main subtypes of DM are type1 diabetes mellitus (T1DM) and type 2 diabetes mellitus (T2DM). Several studies have found bidirectional associations between ADHD and DM. In a longitudinal study of all diabetic children in Germany/Austria between 2003 and 2015, the percentage of ADHD in patients with T1DM ranged from 1.91 to 2.93% (9). The study compared patients with T1DM with and without ADHD and found that ammonia and poor glycemic control were significantly higher in diabetic patients with ADHD (10). Furthermore, Akmatov et al. reported that children with ADHD were more likely to be diagnosed with T1DM and T2DM (4). Therefore, investigating the comorbidity of ADHD and DM is important for the early identification of children at risk for DM or ADHD to ensure that they receive prompt and appropriate treatment. This study aimed to conduct a systematic review and meta-analysis of studies that assessed the prevalence of ADHD in DM patients as well as the prevalence of DM in ADHD patients compared with hose without ADHD. Furthermore, we explored the impacts of ADHD on glycemic control in patients with DM.

## Materials and methods

This study was performed according to the PRISMA guidelines, the Cochrane Handbook for Systematic Reviews, and the Ottawa Non-Randomized Studies Workshop (11–13).

### Search strategy

The search strategy was designed to address the following question: “Is there an interrelationship between ADHD and DM?” The search was completed on November 23, 2021, using the following electronic databases: PubMed, Ovid EMBASE (1947 to present), Web of Science, and PsychInfo. The search terms included “DM”, “T1DM”, “T2DM”, “attention-deficit disorder”, “attention deficit”, and “hypersensitivity” (see Supplementary material). The references of related review articles and all included studies were manually searched for additional relevant studies. The literature search was limited to articles published in English.

### Study selection and eligibility criteria

Two authors (YA and TZ) conducted the study selection, and any disagreement was settled by discussion with a third author (JZ). A flow diagram is provided in Supplementary material. We first assessed eligibility by screening the abstracts and titles of the studies. The remaining studies were read in full text to decide whether to include or exclude the study in accordance with the eligibility criteria listed below.

Observational studies (cohort, cross-sectional, or case-control studies) that reported the prevalence of diabetes among patients with ADHD or that reported ADHD among diabetes patients were considered eligible for this study. Studies that compared glycemic control in patients with DM with and without ADHD were also included. The indexes of glycemic control were hemoglobin A1c (HbA1c) level, frequency of hypoglycemia, and ketoacidosis. Eligibility was not limited by the type of DM. Studies that included neonates, studies without distinguishable comparison groups, and studies that did not report prevalence data or provide original data to calculate were excluded. If more than one study reported the same outcome indexes using data from the same study subjects, the study with more comprehensive results was selected.

### Data extraction and risk of bias

We extracted the following information from the included studies: first author names, publication year, country, study design, sample size, participants’ age, ADHD assessment, and diabetes assessment. For studies reporting the prevalence of ADHD or diabetes, data sufficient to complete a 2 × 2 contingency table and any reported adjusted prevalence odds ratios (ORs) were extracted. For studies comparing glycemic control, we extracted HbA1c (means and standard deviations), and the presence of acute complications, such as diabetic ketoacidosis (DKA) or hypoglycemic attacks.

We employed the Newcastle-Ottawa Scale (NOS) to evaluate each eligible study (14). This scale determines the study quality based on three aspects: selection, comparability, and exposure assessment. Each study was assessed using a NOS score from 0 to 9, and a score greater than seven was deemed “high quality”. Data extraction and quality assessment were conducted independently by two authors (YA and TZ), and disagreements were discussed with a third author (JZ) to reach a consensus.

### Statistical analysis

Because apparent differences in characteristics (e.g., study design, sample size, exposure assessment, and outcomes) existed among the included studies, we only calculated the pooled prevalence estimate using ORs and 95% confidence interval (CI) based on the random-effects model, as recommended by the Ottawa Non-Randomized Studies Workshop (15). A second meta-analysis was conducted to explore the research question (“Are there differences in glycemic control in DM individuals with ADHD and without ADHD?”). The data on the prevalence of hypoglycemia and DKA were entered into dichotomous formats, and then the random effects model was used to calculate the pooled ORs. The HbA1C levels were entered into continuous formats and were obtained for the pooled standardized mean of difference (SMD). Heterogeneity was assessed using both I^2^ statistics and the Q-test method (16). If I^2^ ≥ 50% or the P value for the Q-test was <0.01, then the heterogeneity was considered “high.” The degree of publication bias was assessed by visually inspecting funnel plots, in which the logORs were plotted against their standard errors. There was insufficient data to carry out the planned meta-regression and subgroup analyses. All the above statistical analyses were performed using the Review Manager Software version 5.3.

## Results

### Description of included studies and risk of bias

The systematic literature search and study selection details are shown in the Supplementary material (Diagram 1). A total of 386 non-duplicate studies were retrieved from the databases and the reference lists of the review. After screening the titles and abstracts, 32 studies were read in full. After additional evaluation, 15 studies were excluded because they did not report the ADHD or DM outcome, case series of neonates with diabetes, and reporting scores of ADHD scales in T1DM patients without a comparison group. A total of 17 articles were included in our data extraction (4, 9, 10, 17–30). Among the included studies, three studies (17, 18, 30) had overlapping populations reporting different types of diabetes in children or adults, so we included all of them. Of the eligible studies, five were cohort studies, three were case-control studies, and nine were cross-sectional studies (Table 1). These studies were carried out in Germany, Australia, Sweden, Taiwan, Turkey, and Israel with various data sources. Among these studies, five studies used data linked to a health insurance database (4, 17, 18, 20, 30), seven studies collected data from hospital patients (9, 10, 19, 25, 26, 28, 29), and the remaining five studies collected data from national health research databases. The majority of studies examined children or adolescents, whereas only two were examined adults. Glycemic control was only reported in T1DM patients in nine studies (9, 10, 24–30). All studies failed to report the proportion of responders. Three studies failed to report the original total number of cases and controls (4, 20, 30). One study relied upon in-person household interviews to identify cases of ADHD and/or diabetes (22). Eight studies were adjusted for potential confounders (4, 12–19, 21–23, 30). None of the included studies described the drug therapy of ADHD, and severity of ADHD.

**TABLE 1:** Study characteristics.

Study ID	Country	Study design (Period)	Sample size	Age (years)	Diabetes types	Diagnosis of ADHD	Diagnosis of diabetes
Akmatov et al., (4)	Germany	Case-control (2017)	ADHD = 258662 controls = 2327958	5–14	T1DM T2DM	Diagnosed according to ICD-10 code by data on ambulatory claims	Diagnosed according to ICD-10 code by data on ambulatory claims
Chen et al., (17)	Taiwan	Case-control (2002–2008)	ADHD = 4302 controls = 21510	5–15	T1DM T2DM	Diagnosed according to ICD-9-CM code by psychiatrists	Diagnosed according to ICD-9-CM code by doctor
Chen et al., (18)	Taiwan	Cohort (2002–2011)	ADHD = 35949 controls = 71898	10–29	T2DM	Diagnosed according to ICD-9-CM code by psychiatrists	Diagnosis of DM given by doctors, internal medicine physicians, endocrinologists and family medicine physicians based on the laboratory examination
Nielsen et al., (19)	Denmark	Cohort (1995–2012)	*N* = 983680 ADHD = 23645	5–15	T1DM	Admitted to a psychiatric hospital or had received outpatient care with a diagnosis of ADHD	Admitted or been in outpatient care with diabetes
Kapellen et al., (20)	Germany	cross-sectional (2014)	T1DM = 9654 controls = 3525678	< 18	T1DM	Diagnosed according to ICD-10 code by psychiatrists or treatment with ADHD medication	Diagnosed according to ICD-10 code by psychiatrists or insulin treatment
Butwicka et al., (21)	Sweden	cohort (1973–2009)	T1DM = 17122 controls = 1696611	< 18	T1DM	Diagnosed according to ICD(8-10) code by register data	Diagnosed according to ICD(8-10) code by register data
Xu et al., (22)	USA	cross-sectional (2007–2012)	ADHD = 1642 controls = 51179	20–79	diabetes mellitus	In-person household interview (told by a doctor)	In-person household interview (told by a doctor)
Chen et al., (23)	Sweden	cross-sectional (2013)	ADHD = 61129 controls = 5490678	18–64	T2DM	Diagnosed according to ICD(9-10) code by register data	Diagnosed according to ICD(9-10) code by register data
Hilgard et al., (9)	Germany and Austria	cross-sectional (2003–2015)	*N* = 56722 ADHD = 160880	< 20	T1DM	Reported by the families or based on psychologic or psychiatric evaluation	Based on medical records
Sakhr et al., (10)	Egypt	case-control (2018–2019)	ADHD = 20 controls = 40	6–18	T1DM	Diagnosed according to DSM –IV by psychiatrist	Based on medical records
Vinker-Shuster et al., (24)	USA	cross-sectional (2016)	*N* = 230 ADHD = 24	5–18	T1DM	Based on medical records and using at least 3 prescriptions for ADHD treatment	Based on medical records
Macek et al., (25)	Slovenia	cross-sectional (2013–2014)	*N* = 101 ADHD = 12	11–17	T1DM	Diagnosed according to DSM –IV by psychiatrist	Based on medical records
Nylander et al., (26)	Sweden	cross-sectional (2013–2015)	*N* = 166 ADHD = 16	12–18	T1DM	Diagnosed according to ADHD RS-IV by psychiatrist	Based on medical records
Liu et al., (27)	Sweden	Cohort (1973–2013)	*N* = 11326 ADHD = 514	< 18	T1DM	Diagnosed according to ICD (9–10) code by register data or prescribed with ADHD medication	Based on register data
Yazar et al., (28)	Turkey	cross-sectional (2017)	*N* = 61 ADHD = 15	< 19	T1DM	Diagnosed according to ADHD RS-IV by psychiatrist	Based on medical records
Mazor-Aronovitch et al., (29)	Israel	cross-sectional (2011)	*N* = 121 ADHD = 39	6–18	T1DM	Diagnosed according to DSM –IV and ADHD-RS by psychiatrist	Based on medical records
Lin et al., (30)	Taiwan	Cohort (1998–2011)	*N* = 3875 ADHD = 726	≤ 18	T1DM	Diagnosed according to ICD-9-CM code by psychiatrists	Diagnosed according to ICD-9-CM code by health insurance data

ADHD, attention deficit hyperactivity disorder; T1DM, Type 1 diabetes mellitus; T2DM, Type 2 diabetes mellitus.

### Prevalence estimates

**FIGURE 1:**
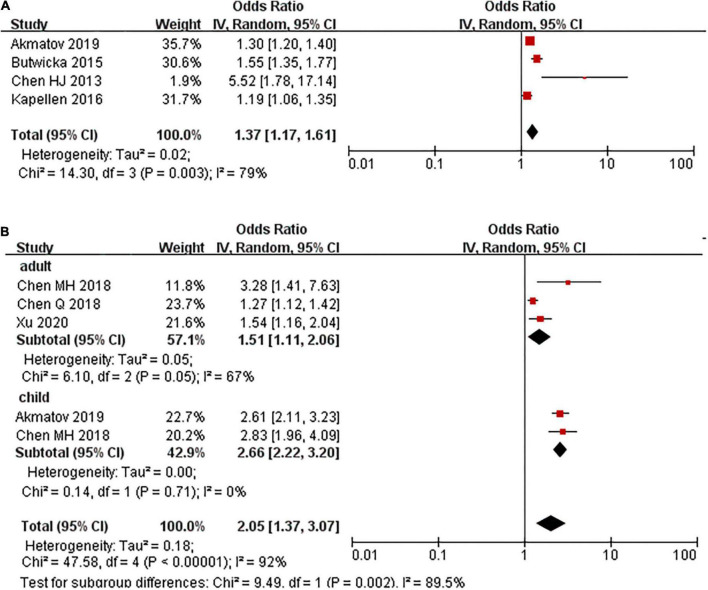
Prevalence ratio of T1DM and T2DM in people with ADHD, stratified on age group. **(A)** T1DM. **(B)** T2DM.

Four studies examined the risk of ADHD in children with T1DM compared with children without T1DM. There was a 35% increase in the prevalence of ADHD among children with T1DM compared with those without T1DM (OR: 1.35, 95% CI 1.08–1.73, *I*^2^ = 64%, *p* = 0.04) (Supplementary Figure 1). The pooled estimate from the other four studies also showed an increased risk of T1DM in children with ADHD (OR: 1.37, 95% CI 1.17–1.61, *I*^2^ = 79%, *p* < 0.01; Figure 1).

None of the studies have assessed the risk of ADHD in patients with T2DM. Five studies evaluated the risk of T2DM in patients with ADHD compared to those without ADHD. The pooled results showed that a higher proportion of patients in the ADHD group had T2DM compared with the control group (OR: 2.05, 95% CI: 1.37–3.07, *I*^2^ = 92%, *p* < 0.01; Figure 1). The high degree of heterogeneity between the studies may be explained by the age group. In those studies where participants were under 18 years old, the pooled PR was 2.66 (2.23, 3.20), where it was 1.51 (1.11, 2.06) in adults (Figure 1).

### Mean differences of hemoglobin A1c

**FIGURE 2:**
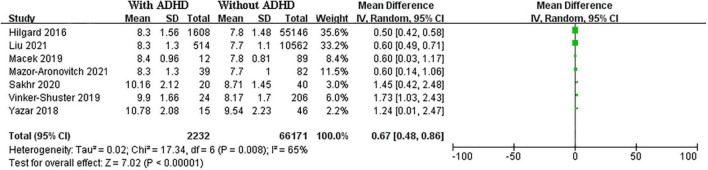
Forest plots of studies showing standardized mean difference for HbA1C level in in T1DM patients with and without ADHD.

A total of seven studies including 2,232 cases and 6,6171 controls were pooled for HbA1C levels. The SMD of HbA1C was 0.67 (95% CI: 0.48–0.86 *I*^2^ = 65%, *p* < 0.01) between T1DM patients with ADHD and those without ADHD (Figure 2).

### Hypoglycemia and diabetic ketoacidosis

**FIGURE 3:**
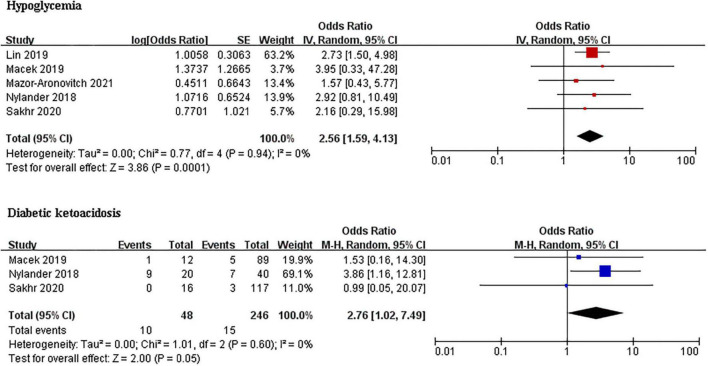
Forest plots of studies for comparing the prevalence of hypoglycemia and DKA in T1DM patients with and without ADHD.

Type 1 diabetes mellitus (T1DM) patients with ADHD had a significantly higher prevalence of hypoglycemia than those without ADHD (OR 2.29, 95% CI: 1.04–5.03, *I*^2^ = 0%; *p* = 0.88). Pooled DKA was performed in 48 cases and 246 controls in (Figure 3). The pooled analysis for the DKA prevalence found that T1DM patients with ADHD were also more likely to have DKA than those without ADHD (OR 2.76, 95% CI: 1.02–7.49, *I*^2^ = 0%, *p* = 0.60).

### Publication bias

The asymmetric shapes of the funnel plots indicated the existence of publication bias in the selected studies.

## Discussion

To our knowledge, this is the first study to evaluate and consolidate the literature on the association between ADHD and diabetes. Significant heterogeneity was identified between the prevalence estimate and mean difference studies. The population of interest, sample size, and adjustment for potential confounders appeared to have been an important source of heterogeneity. Our pooled analyses suggest an important comorbid relationship between diabetes and ADHD. Overall, there was an increase in T2DM of 166% and 51% in children and adults with ADHD, respectively, relative to those without ADHD. An overall 37% increase in T1DM was observed in children with ADHD. In addition, 35% of the children with T1DM also met the criteria for ADHD.

The results from the second meta-analysis in T1DM patients detected an overall pooled SMD in HbA1C levels between children with ADHD and those without ADHD. Despite the high level of heterogeneity among these studies, no further subgroup analysis was conducted owing to the small number of studies included. Further analyses identified higher hypoglycemia and DKA index rates within the ADHD groups compared with their non-ADHD counterparts.

The mechanisms underlying the association between diabetes and ADHD are unclear. Most studies have focused on the prevalence of T2DM and neurologic diseases among middle-aged and elderly individuals, and there is little information available regarding this effect among children and adolescents. Diabetes is a group of metabolic syndromes characterized by hyperglycemia. The disturbance of blood glucose can lead to a series of pathogenetic mechanisms involving neurological dysfunction, including neuronal apoptosis, abnormal energy metabolism, synaptic dysfunction, neurodegenerative changes, and oxidative stress in brain tissue (31–34). Compared with healthy controls, T1DM patients showed fractional anisotropy values in major association fibers of the frontal, parietal, and temporal regions 20 years after diagnosis (35). Cognitive impairment in T2DM has also been found to be related to reduced activation in the hemisphere temporoparietal regions (36). Damage to these brain areas may lead to memory deficits, deficit in executive functions, inattention, and loss of emotion. Diabetic patients have various metabolic indexes in addition to blood glucose disorders. Disorders of lipid metabolism and increased levels of ghrelin, cortisol, and C-reactive protein can also lead to a decrease in neurological function (37, 38). Research at the gene level may also help to explain the association between ADHD and diabetes. In an analysis using summary-level data from previous genome-wide association studies, significant positive relationships were observed between ADHD and psoriasis, rheumatoid arthritis, and tuberculosis susceptibility (39). In patients with T2DM, The single nucleotide polymorphisms rs17518584 and Hp1-1 in patients with T2DM are associated with executive functions and attention/working memory, respectively (40, 41). Future studies are needed to elucidate these mechanisms and identify drug targets.

Several factors may be involved in the association between ADHD and diabetes. Evidence emerging supports the role of maternal diabetes in neurocognitive and behavioral difficulties in their offspring, including ADHD (42–44). Exposure to maternal pregestational diabetes (OR and 95%CI 1.4 1.31–1.50) or preexisting type 1 diabetes (OR and 95%CI 1.39 1.27–1.52) increased the risk of ADHD in offspring (44). In addition, maternal diabetes (all types) increases the risk of developing childhood-onset T1DM (45). But none of the included studies provided the results adjusted for maternal diabetes, and future studies should take note of the similar risk study of ADHD and DM. Obesity is a strong and independent risk factor for diabetes. In a recent meta-analysis of 95 studies, the pooled estimates of the prevalence of obesity and being overweight were 14.7%, and 20.9%, respectively, in individuals with ADHD (5). However, Chen et al. (17) and Xu et al. (22) reported that the association between ADHD and diabetes in adults and children persisted after additional adjustment for obesity and BMI, indicating that there might be other mechanisms linking ADHD and diabetes.

Furthermore, we found patients with T1DM and ADHD had poor glycemic control. There may be many ways ADHD impacts an individual’s ability to control diabetes. Previous studies have shown that typical symptoms of ADHD not only lead to a higher risk of DKA but also increase the risk of severe hypoglycemia (46, 47). The mismanagement of insulin therapy is important reason for glycemic control in T1DM patients with comorbid ADHD (48, 49). It is also increasingly recognized that antipsychotic medications have multiple adverse effects on weight, lipids and glucose metabolism and cardiovascular disease (50). Another explanation for glycemic disorder might be missed insulin injections due to lack of attention and/or impulsiveness, leading to uncontrolled eating habits (49). Although more research is needed to address these questions, our study suggests that this group requires special care and attention from the medical staff. Unique measures such as prescribed, change in eating habit, and creation of Apps are needed to overcome limitations in diabetes management in children with comorbid ADHD.

This meta-analysis has certain limitations. First, the majority of the included studies were not cohort studies. Based on the evidence gathered, we could only speculate on the possible coexisting conditions between ADHD and DM; the onset and the causative pathways between the two conditions could not be identified. Second, we only included studies published in English. The number of studies and sample size were insufficient. We could not perform a subgroup or meta-regression analysis to determine whether the association may or may not be influenced by other factors. Our study’s findings should be interpreted in light of statistical analysis limitations in a small sample. Third, marked heterogeneity was observed across the results of the included studies. This may be due to differences in population selection, the sample sizes of the studies, and outcome measure assessments. For example, five studies (4, 17, 18, 20, 30) collected data from a health insurance database. The possibility of misclassifying of ADHD cases or comorbid metabolic disorders cannot be ruled out. In terms of the mean HbA1c, Macek et al. (25) reported the data for the preceding 12 months, and Liu et al. calculated the area under the curve divided by the time interval between the first and last recorded HbA1c (27). Fourth, the identified studies were controlled irregularly for confounding factors. Most studies provided adjusted results for controlling for age and sex, but they generally lacked data on the parental history of diabetes or mental disorders, lifestyle factors, and socioeconomic status, which are important risk factors for diabetes. In addition, included studies lacked the medicine prescription and severity of ADHD. Finally, the degree of metabolic disorder confounders (e.g., obesity and hypertension) in the association between ADHD and DM remains unknown.

## Conclusion

This systematic review and meta-analysis evaluated studies exploring the association between ADHD and DM. It is important for doctors who treat people with ADHD or DM to be aware of the comorbid association between ADHD and DM. Our study suggests that children with T1DM and ADHD are more likely to have poor glycemic control than those without ADHD. Furthermore, Liu et al. suggested an increased risk of diabetic nephropathy and nephropathy in patients with T1DM and ADHD (27). This association also implicates a potential intervention for both ADHD and DM. Further studies are needed to better understand the relationship between ADHD and DM. If this association is different in various age groups (children and adults), the type of DM should be considered. To improve the quality of evidence, controlling for potential confounders, especially obesity, BMI, prescription for ADHD, and other potential coexisting mental diseases (e.g., anxiety disorder, autism spectrum disorder, depression), should also be a priority.

## Data availability statement

The original contributions presented in this study are included in the article/Supplementary material, further inquiries can be directed to the corresponding authors.

## Author contributions

YA and TZ: conception of the work, literature search, and drafting the article. YA, TZ, and HL: data analysis. JL and HL: critical revision of the article. All authors final approval of the version to be published.

## Abbreviations

DM: diabetes mellitus
ADHD: attention deficit hyperactivity disorder
T1DM: type1 diabetes mellitus
T2DM: type 2 diabetes mellitus
OR: odds ratios
DKA: diabetic ketoacidosis
NOS: Newcastle-Ottawa Scale
SMD: standardized mean of difference
CI: confidence interval.
