# Supplementary material for: The relationship between diabetes mellitus and attention deficit hyperactivity disorder: A systematic review and meta-analysis
Source: Front Pediatr. 2022 Sep 29;10:936813. doi: 10.3389/fped.2022.936813 (PMC9560781; doi:10.3389/fped.2022.936813)
Supplement: Supplementary file 1 [file Data_Sheet_1.docx]

**TITLE** The relationship between diabetes mellitus and attention deficit hyperactivity disorder: a systematic review and meta-analysis

**Contents**

Diagram 1. Flow chart of the literature search and selection process------------page 2

Search strategy--------------------------------------------------------------------------page 3

sTable 1. Raw data of the prevalence of ADHD and diabetes -------------------page 4

sTable 2-4. Raw data of HbA1c level, prevalence of hypoglycaemia and DKA-- page 5

Quality score of included studies--------------------------------------------------- page 6-7

sFigure 1. The prevalence of ADHD children with T1DM and controls------ page 8

**Diagram 1. Flow chart of the literature search and selection process**

## Identification

## Eligibility

## Included

## Screening

Additional records identified through other sources
(n = 21)

Records identified through database searching
PubMed (n = 351) Web of Science (n = 398) EMBASE (3429) PsycInfo (n = 96)

Records from study search
(n =4295)

Records excluded for duplicated studies
(n =674)

Records screened
(n =3621)

Records excluded for clearly irrelevant, review, case reports
(n = 3589)

Full-text articles assessed for eligibility
(n = 32)

Full-text articles excluded, with reasons (n = 15)

No ADHD data (8)

No diabetes data (5)

No comparion group (2)

Overlapping populations (2)

Studies included in quantitative synthesis (meta-analysis)
(n = 17)

**Electronic database search strategy**

**Pubmed**

**Limit to Human and English**

("Diabetes Mellitus"[MeSH Terms] OR "Diabetes Mellitus, Type 1"[Mesh] OR "Diabetes Mellitus, Type 2"[Mesh] OR "diabetes*"[All Fields] OR "T1DM*"[All Fields] OR "T2DM*"[All Fields]) OR " glycated haemoglobin*"[All Fields]) AND ("Attention Deficit Disorders with Hyperactivity"[MeSH Terms] OR" Attention Deficit Hyperactivity Disorder"[ All Fields] OR " Hyperkinetic Syndrome"[ All Fields] OR "ADHD"[All Fields] OR "attention deficit disorder "[All Fields])

**PsycInfo**

((“diabetes mellitus” OR “diabetes” OR “insulin”) AND (“Attention Deficit Disorder with Hyperactivity” OR “ADHD” OR “Hyperactivity” OR “hyperkinetic”) AND (“prevalence” OR “population” OR “incidence”))

**Web of Science**

**Limit to Human and English**

((“diabetes” OR “diabetes mellitus” OR diabete*) AND (“Attention Deficit Disorder" OR ADHD OR hyperactiv* OR impulsiv*) AND (prevalence OR incidence OR epidemiolog* OR population OR community))

**Ovid EMBASE**

**Limit to Human and English**

((“Diabetes Mellitus”/exp OR “Diabetes”/exp OR diabete* OR type 2 diabete* OR type 1 diabete *) AND (“Attention Deficit Disorder”/exp OR “Attention Deficit Disorder with Hyperactivity”/exp OR hyperactiv* OR impulsiv* OR hyperkinetic*) AND (“Epidemiology”/exp OR “Population”/exp OR prevalence OR incidence OR epidemiolog* or population OR community))

| **sTable 1. Raw data of included studies reporting the prevalence of ADHD and diabetes** | | | | | | |
| --- | --- | --- | --- | --- | --- | --- |
| **Study** | **A^+^ D^+^** | **A^+^ D^-^** | **A^-^ D^+^** | **A^-^ D^-^** | **Adjusted Results (95%CI)** | **Adjustment for possible confounders** |
| Akmatov 2019 [4] | NR | NR | NR | NR | Risk of DM in ADHD  T1DM 1.30 (1.20-1.40)  T2DM 2.61 (2.11-3.23) | gender, age, and region of residence |
| Butwicka 2015 [21] | 211 | 13575 | 16911 | 1683036 | Risk of ADHD in T1DM  1.5 (1.3–2.7) | age, sex, year, county, maternal/paternal age at childbirth, maternal/paternalpsychiatric history, maternal/paternal country of birth, level of education, gestational age, birthweight, being born small for gestational age, being born large for gestational age, Apgar score, and history of psychiatric disorders prior to therecruitment |
| Chen HJ 2013 [17] | 4 | 4298 | 12 | 21498 | Risk of ADHD in T1DM  1.62 (0.51–5.14) | age, sex, index year, geographic location, and obesity |
| Chen HJ 2013 [17] | 36 | 4266 | 64 | 21446 | Risk of ADHD in T1DM  2.75 (1.82–4.16) | age, sex, index year, geographic location, and obesity, |
| Chen MH 2018 [18] | 158 | 35791 | 79 | 71819 | Risk T2DM in ADHD  < 18y 2.83(1.96-4.09)  18-29y 3.28(1.41-7.63) | Age, sex, level of urbanization, income, use of ADHD medications, atypical antipsychotics, hypertension, dyslipidemia, obesity |
| Kapellen 2016 [20] | 276 | 84867 | 9378 | 3440811 | NR | NR |
| Nielsen 2017 [19] | NR | NR | NR | NR | Risk of ADHD in T1DM  1.31 (1.03,1.63) | calendar year, sex, and its interaction with age, paternal history of psychiatric admission. |
| Xu 2020 [22] | 115 | 1527 | 4516 | 46663 | Risk of DM in ADHD  1.54 (1.16-2.04) | age, sex, race/ethnicity, education, family income level, alcohol drinking, smoking, and physical activity, BMI |
| Chen Q 2018 [23] | 253 | 4611 | 58958 | 1602116 | Risk of T2DM in ADHD  1.27 (1.12-1.42) | sex and age in years |

NR = not reported; A^+/-^ = people with or without ADHD; D^+/-^ = people with or without diabetes mellitus.

| **sTable 2. Raw data of included studies reporting the HbA1c in T1DM** | | | | | | |
| --- | --- | --- | --- | --- | --- | --- |
| **Study** | **A^+^ D^+^** | | | **A^-^ D^+^** | | |
| HbA1c (%) | Mean | SD | Number | Mean | SD | Number |
| Hilgard 2016[9] | 8.3 | 1.56 | 1608 | 7.8 | 1.48 | 55146 |
| Vinker-Shuster 2019[24] | 9.9 | 1.66 | 24 | 8.17 | 1.7 | 206 |
| Macek 2019[25] | 8.4 | 0.96 | 12 | 7.8 | 0.81 | 89 |
| Sakhr 2020[10] | 10.16 | 2.12 | 20 | 8.71 | 1.45 | 40 |
| Yazar 2018[28] | 10.78 | 2.08 | 15 | 9.54 | 2.23 | 46 |
| Mazor-Aronovitch 2021[29] | 8.3 | 1.1 | 39 | 7.7 | 1.0 | 82 |
| Liu 2021[27] | 8.3 | 1.3 | 514 | 7.7 | 1.1 | 10562 |

| **sTable 3. Raw data of included studies reporting the prevalence of hypoglycaemia in T1DM** | | | | |
| --- | --- | --- | --- | --- |
| **Study** | **A^+^ D^+^** |  | **A^-^ D^+^** |  |
| Hypoglycemia | Events | Number | Events | Number |
| Macek 2019[25] | 1 | 12 | 2 | 89 |
| Sakhr 2020[10] | 5 | 20 | 7 | 40 |
| Nylander 2018[26] | 4 | 16 | 12 | 117 |
| Mazor-Aronovitch 2021[29] | 2 | 39 | 2 | 82 |

| **sTable 4. Raw data of included studies reporting the prevalence of DKA in T1DM** | | | | |
| --- | --- | --- | --- | --- |
| **Study** | **A^+^ D^+^** | | **A^-^ D^+^** | |
| DKA | Events | Number | Events | Number |
| Macek 2019[25] | 1 | 12 | 5 | 89 |
| Sakhr 2020[10] | 9 | 20 | 7 | 40 |
| Nylander 2018[26] | 0 | 16 | 3 | 117 |
| Lin 2019 [30] | Only provided adjusted risk of ADHD in hypoglycaemia patients 2.73 (1.50-4.98) | | | |

A+/- = people with or without ADHD; D+/- = people with or without diabetes mellitus.

**The Detailed Assessment Process of Every Included Article (NOS)**

|  |  |  |  |  |  |  |  |  |  |  |
| --- | --- | --- | --- | --- | --- | --- | --- | --- | --- | --- |
| Cohort study | | Selection 1) | Selection 2) | Selection 3) | Selection 4) | Comparability 1) | Outcome 1) | Outcome 2) | Outcome 3) | Score |
| Chen MH 2018 [18] | | b) | a) | a) | a) | a) | b) | b) | d) | 6 |
| Nielsen 2017[19] | | a) | a) | a) | a) | a) | b) | a) | d) | 7 |
| Butwicka 2015[21] | | a) | a) | a) | a) | a) | b) | a) | d) | 7 |
| Liu 2021 [27] | | a) | a) | a) | a) | - | b) | a) | d) | 6 |
| Lin 2019 [30] | | a) | a) | a) | a) | a) | b) | a) | d) | 7 |

|  |  |  |  |  |  |  |  |  |  |  |
| --- | --- | --- | --- | --- | --- | --- | --- | --- | --- | --- |
| Case-control study | | Selection 1) | Selection 2) | Selection 3) | Selection 4) | Comparability 1) | Exposure 1) | Exposure 2) | Exposure 3) | Score |
| Akmatov 2019[4] | | a) | a) | a) | a) | - | a) | a) | b) | 6 |
| Chen HJ 2013 [17] | | a) | a) | b) | a) | a) | a) | a) | b) | 6 |
| Sakhr 2020 [10] | | a) | a) | b) | a) | a) | a) | a) | b) | 5 |

| Cross-sectional study | Selection 1) | Selection 2) | Selection 3) | Selection 4) | Comparability  1) | Outcome  1) | Outcome 1) | Outcome 2) | Score |
| --- | --- | --- | --- | --- | --- | --- | --- | --- | --- |
| Kapellen 2016[20] | b) | a) | c) | a) | - | b) | a) | a) | 7 |
| Xu 2020 [22] | a) | a) | c) | b) | a) | c) | a) | b) | 5 |
| Chen Q 2018 [23] | a) | a) | c) | a) | a) | b) | a) | a) | 7 |
| Hilgard 2016 [9] | a) | a) | c) | b) | a) | b) | a) | a) | 7 |
| Macek 2019 [25] | b) | a) | c) | a) | - | b) | a) | a) | 7 |
| Mazor-Aronovitch 2021[29] | b) | a) | c) | a) | - | b) | a) | a) | 7 |
| Nylander 2018 [26] | b) | a) | b) | a) | - | b) | a) | a) | 7 |
| Yazar 2018 [28] | b) | a) | c) | a) | - | c) | a) | a) | 6 |
| Vinker-Shuster 2019 [24] | b) | a) | c) | a) | - | b) | a) | a) | 7 |

**sFigure 1. The prevalence of ADHD children with T1DM and controls**

**

**
